# A Kunitz-type inhibitor from tick salivary glands: A promising novel antitumor drug candidate

**DOI:** 10.3389/fmolb.2022.936107

**Published:** 2022-08-16

**Authors:** Aline R. M. Lobba, Miryam Paola Alvarez-Flores, Melissa Regina Fessel, Marcus Vinicius Buri, Douglas S. Oliveira, Renata N. Gomes, Priscila S. Cunegundes, Carlos DeOcesano-Pereira, Victor D. Cinel, Ana M. Chudzinski-Tavassi

**Affiliations:** ^1^ Centre of Excellence in New Target Discovery-CENTD, Butantan Institute, São Paulo, Brazil; ^2^ Development and Innovation Centre, Butantan Institute, Butantan Institute, São Paulo, Brazil; ^3^ Biochemistry Department, Federal University of São Paulo, São Paulo, Brazil

**Keywords:** amblyomin-X, proteasome inhibitor, TFPI-like, antitumor / cytotoxic activity, bioactive molecule

## Abstract

Salivary glands are vital structures responsible for successful tick feeding. The saliva of ticks contains numerous active molecules that participate in several physiological processes. A Kunitz-type factor Xa (FXa) inhibitor, similar to the tissue factor pathway inhibitor (TFPI) precursor, was identified in the salivary gland transcriptome of *Amblyomma sculptum* ticks. The recombinant mature form of this Kunitz-type inhibitor, named Amblyomin-X, displayed anticoagulant, antiangiogenic, and antitumor properties. Amblyomin-X is a protein that inhibits FXa in the blood coagulation cascade and acts via non-hemostatic mechanisms, such as proteasome inhibition. Amblyomin-X selectively induces apoptosis in cancer cells and promotes tumor regression through these mechanisms. Notably, the cytotoxicity of Amblyomin-X seems to be restricted to tumor cells and does not affect non-tumorigenic cells, tissues, and organs, making this recombinant protein an attractive molecule for anticancer therapy. The cytotoxic activity of Amblyomin-X on tumor cells has led to vast exploration into this protein. Here, we summarize the function, action mechanisms, structural features, pharmacokinetics, and biodistribution of this tick Kunitz-type inhibitor recombinant protein as a promising novel antitumor drug candidate.

## Introduction

Historically, natural products extracted from plants, herbs, animals, and microorganisms have been a reliable source of bioactive molecules for pharmaceutical discovery. Despite this complexity, recent technological advances and the advent of new methods for high-throughput screening have contributed to the resurgence of pharmaceutical interest in natural products or their direct derivatives for developing new drugs (Nogueira et al., 2010; Brahmachari, 2011; Harvey et al., 2015). Plant extracts have traditionally been used as a source of potential compounds, but animal-derived drugs have also been crucial in several diseases. There is increasing interest in bioactive molecules from other sources, such as arthropods and parasites (Calixto, 2019). Tick salivary glands are recognized as a rich source of pharmaco-active molecules (Chmelař et al., 2019; Aounallah et al., 2020).

Ticks are obligate hematophagous parasites that must overcome their vertebrate host’s sophisticated immune defense systems to feed, and salivary glands are vital structures responsible for their biological success. Tick saliva contains numerous physiologically active molecules that participate in various physiological processes. Crude saliva is a mixture of diverse biomolecules directly involved in blood coagulation, platelet aggregation, vascular contraction, host immunity, and inflammation (Ribeiro and Francischetti, 2003). Previous intensive transcriptomic and proteomic research has identified several protein families from tick salivary glands (Champagne, 2004; Faria et al., 2005; Junqueira-de-Azevedo et al., 2006; Ribeiro et al., 2006; Alarcon-Chaidez et al., 2007; Chmelař et al., 2019). Batista and colleagues (Batista et al., 2008) constructed a cDNA library of the *Amblyomma cajennense*, currently *Amblyomma sculptum* (Nava et al., 2014), salivary gland to search for potential proteins involved in the hemostatic process for drug development.

**FIGURE 1:**
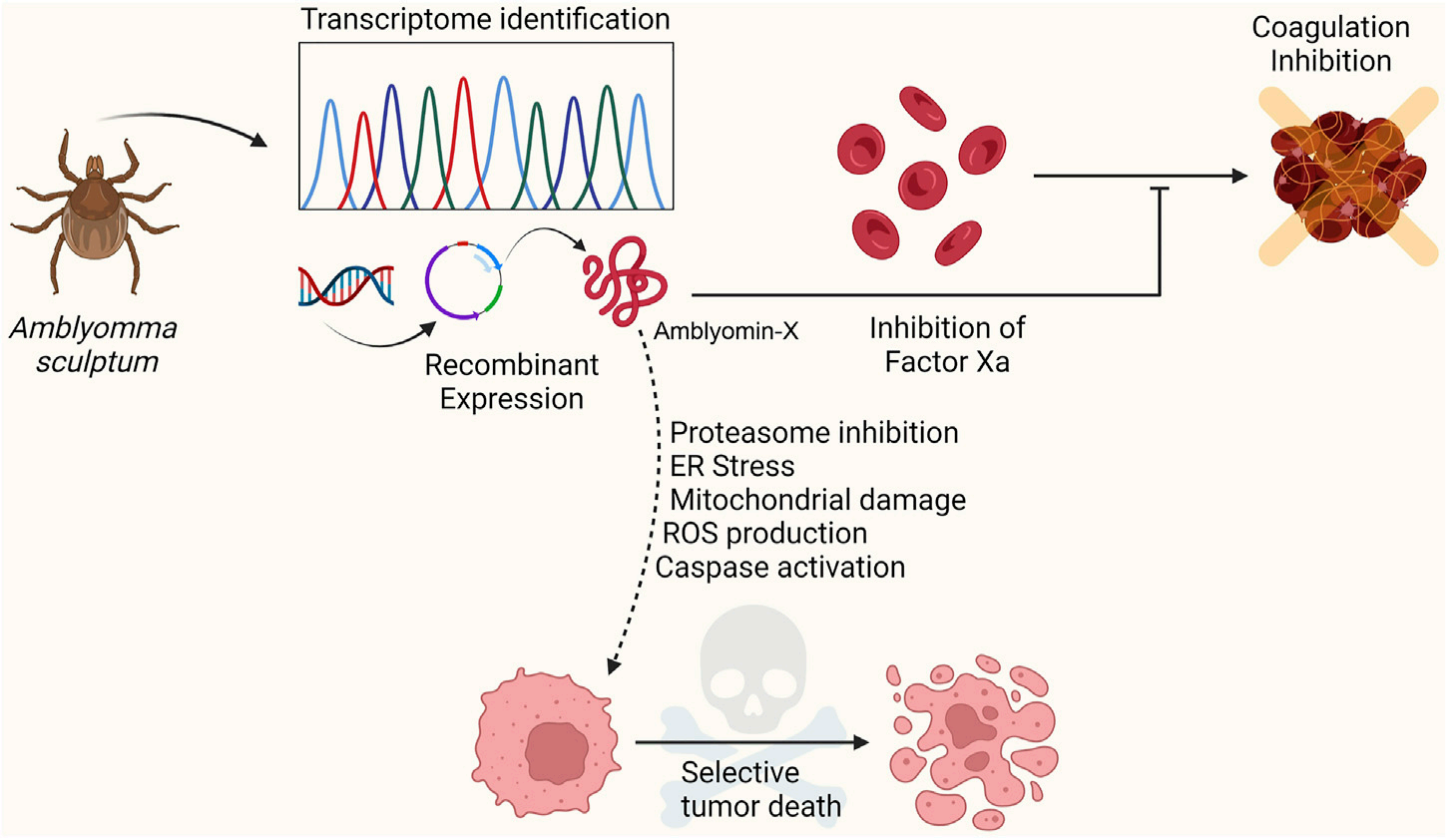
The salivary gland transcriptome of the *Amblyomma sculptum* tick was characterized and analyzed by expressed sequence tags (EST). The study revealed the presence of protein-related transcripts involved in the hemostatic process, especially proteases and inhibitors. A Kunitz-type inhibitor similar to the tissue factor pathway inhibitor (TFPI) precursor was identified. This inhibitor, Amblyomin-X, was obtained as a recombinant protein and presented anticoagulant, antiangiogenic, and antitumor properties. Furthermore, Amblyomin-X demonstrated selectivity for tumor cells. Figure created in BioRender.com.

The gland mRNAs of adult female *A. sculptum* from Brazil were reverse-transcribed to cDNAs and cloned into *Escherichia coli* DH5α cells for large-scale DNA sequencing and expressed sequence tag (EST) generation (Batista et al., 2008). These ESTs were assembled into cluster sequences, searched against the GenBank NCBI database, screened for the presence of potentially full-length open reading frames (ORFs), signal peptides, and conserved domains. The analysis revealed the presence of transcripts related to proteins involved in the hemostatic processes, especially proteases and inhibitors. Batista et al. identified a Kunitz-type protease inhibitor, similar to the tissue factor pathway inhibitor (TFPI) precursor, a physiological factor Xa (FXa) inhibitor, among the protein-related transcripts (Batista et al., 2008; Batista et al., 2010; Mesquita Pasqualoto et al., 2014). The recombinant mature form of this Kunitz-type protease was named Amblyomin-X (Amblyomma Factor Xa inhibitor) and displayed inhibitory activity towards factor X and antiangiogenic and antitumor properties (Figure 1). Here, we summarize the function, molecular mechanisms of action, and structural features of this tick Kunitz-type inhibitor recombinant protein as a promising novel antitumor drug candidate.

## Amblyomin-X structural features

**FIGURE 2:**
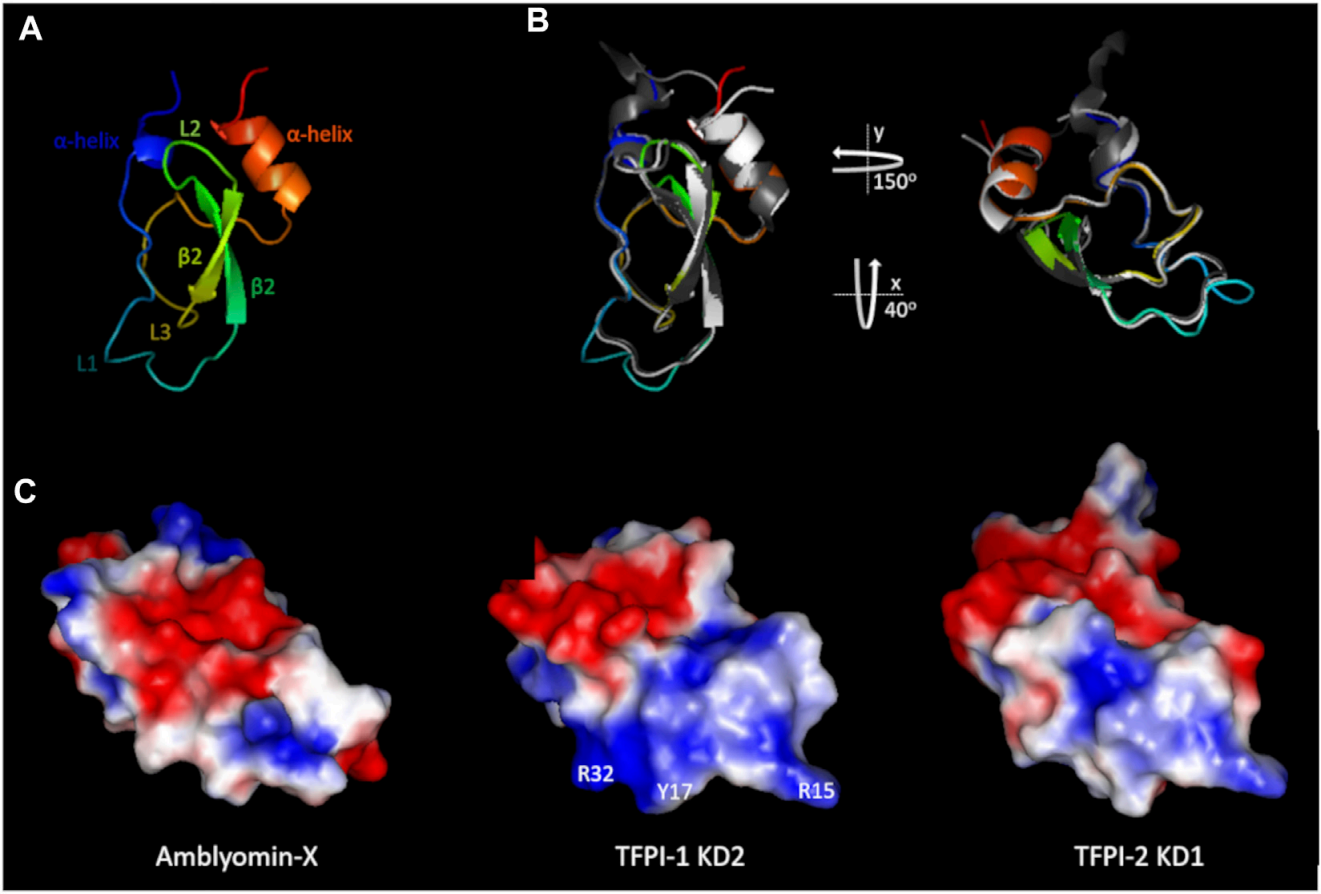
Structural features of Amblyomin-X’s Kunitz domain. **(A)** Rainbow colored Amblyomin-X structure model (blue to red). **(B)** Structure superimposition of the Kunitz domains of Amblyomin-X (rainbow), TFPI-1 KD2 (white), and TFPI-2 KD1 (gray). Structures are shown in two orientations: the same as A (left) and rotated (right) as displayed in **(C) (C)** Electrostatic potential of the molecular surfaces of the indicated Kunitz domains. Some important residues for TFPI-1 KD2 interaction with FXa (Mesquita Pasqualoto et al., 2014) are indicated. Surfaces negatively charged are shown in red, non-charged in white, and positively charged in blue. For Amblyomin-X homology modeling, boophilin (PDB ID: 2ODY) was used as a template. For TFPI-1 KD2 and TFPI-2 KD1, PDB IDs are 1TFX and 1ZR0, respectively.

Amblyomin-X, a protein derived from *Amblyomma sculptum*’s sialotranscriptome analysis (Batista et al., 2008), was named after its bacteria-produced recombinant form, which displayed inhibitory activity towards FXa (Batista et al., 2010; Branco et al., 2016). The identified mRNA sequence (GenBank AY563168.1) encodes a secreted protein (GenBank AAT68575.1) comprising a signal peptide followed by a 58-residues BTPI/Kunitz serine protease inhibitor domain (KD) and a C-terminal sequence with 50-residues, slightly shorter than the KD. Amblyomin-X’s C-terminal contains a unique amino acid sequence that shares no relevant homology with any protein available in public databases. However, the Amblyomin-X KD presents a conserved pattern of six cysteine residues and is expected to assume a disulfide-rich α/β-folded tridimensional organization, characteristic of BPTI/Kunitz domains (Figure 2A).

Amblyomin-X has been reported to inhibit the extrinsic tenase complex and FXa activity (Batista et al., 2010). Therefore, it is reasonable to infer that its KD could be functionally similar to that of TPFI-like inhibitors (Crawley and Lane, 2008). TFPI-1, for instance, is an endogenous tissue factor (TF) inhibitor containing three KDs in tandem, which binds to both FXa (its second KD inhibits FXa) and the extrinsic tenase complex (Crawley and Lane, 2008). In addition, like Amblyomin-X, both TFPI-1 and its structural homolog TFPI-2 present antitumor activities (Crawley and Lane, 2008), although TPFI-2 appears to function as a plasmin inhibitor and only weakly inhibits coagulation (Crawley and Lane, 2008).

Amblyomin-X’s structural model was compared to available three-dimensional structures of the second (TFPI-1 KD2) and first (TFPI-2 KD1) KDs of TFPI-1, and TFPI-2, respectively, to evaluate its relatedness to TFPI-like inhibitors (Mesquita Pasqualoto et al., 2014). As expected, concerning the Kunitz domains, Amblyomin-X, TFPI-1 KD2, and TFPI-2 KD1 present the same overall three-dimensional arrangement: two helixes, an antiparallel *β*-sheet composed of two *β*-strands, and three loops (Figure 2B) (Mesquita Pasqualoto et al., 2014). Although overall protein folding is conserved, it is worth mentioning that Amblyomin-X’s structural visualization revealed a clear difference at loop L1 compared to the other molecules (Figure 2).

The charge distributions at the proteins’ molecular surfaces were inspected because electrostatic interactions are particularly important for protein-protein recognition/interaction (Mesquita Pasqualoto et al., 2014). This analysis revealed that Amblyomin-X presents a distinctive surface charge distribution: while TFPI-1 KD2 and TFPI-2 KD1 share the pattern of negatively charged patches at the upper side and positively charged patches at the bottom. Amblyomin-X presents negative patches at the center and positive patches at the top and bottom (Mesquita Pasqualoto et al., 2014). Identifying some of the TFPI-1 KD2 residues that participate in the FXa inhibitory interaction (Burgering et al., 1997) amplifies the importance of the charge distribution difference between this protein and Amblyomin-X (Figure 2C) (Mesquita Pasqualoto et al., 2014). Several important differences were observed at the bottom of the molecules, particularly in the L1 loop and its vicinity (Figure 2). The predicted trypsin interaction site in this region, a component of the Kunitz domain family, is related to the active site of the inhibitor when it acts *via* a substrate-like mechanism (NCBI Conserved Domain Database CDD:238057).

In this context, it is worth mentioning that even minor alterations in the L1 loop can modify the molecule’s inhibitory ability/specificity, not only in this region. For instance, these differences can, at least in part, help explain why TFPI-2 presents as a distinct inhibitory target from TFPI-1 (Crawley and Lane, 2008).

As reviewed by Corral-Rodríguez et al. (2009) and Blisnick et al. (2017) respectively, z, several other proteins derived from ticks also have been identified as serine protease/coagulation inhibitors, and a number of them are characterized as Kunitz-type inhibitors. Among them, the well studied tick anticoagulant peptide (TAP) identified on extracts of the *Ornithodoros moubata* is a low molecular weight Kunitz-related FXa inhibitor (Waxman et al., 1990); moreover, identified on an *Ixodes scapularis* salivary gland cDNA library, the Ixolaris and Penthalaris, proteins with 2 and 5 tandem Kunitz-like domains, respectively, are able to bind to FXa or FX as a scaffold for inhibition of the TF/FVIIa (Francischetti et al., 2002; Francischetti et al., 2004).

**FIGURE 3:**
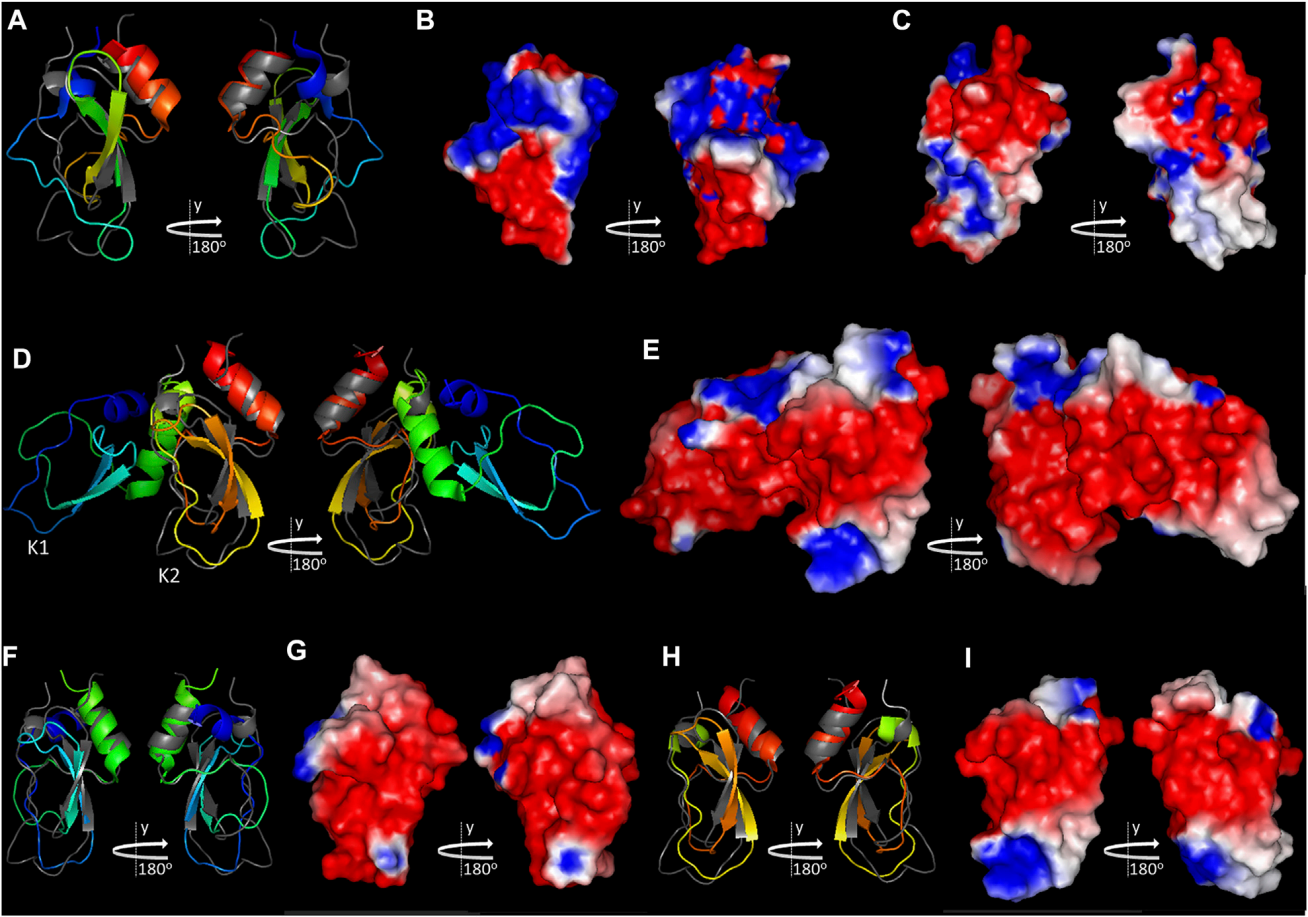
Comparison of Amblyomin-X’s KD with TAP and Ixolaris. Amblyomin-X’s KD homology model (colored gray in A and E, in the same orientation as Figure 2A) was aligned with NMR structures of TAP (PDB: 1TCP) **(A)**, Ixolaris (PDB: 6NAN) **(D)**, and Ixolaris K1 **(F)**, and K2 **(H)**, colored in rainbow (blue to red). Electrostatic potential of the molecular surfaces of **(B)** TAP, **(C)** KD of Amblyomin-X, **(E)** Ixolaris, **(G)** Ixolaris K1, and **(I)** Ixolaris K2 are shown. Structure visualizations were created in PyMOL v.2.5.1.

The tridimensional structures of the recombinant forms from TAP (Lim-Wilby et al., 1995) and Ixolaris (De Paula, et al., 2019) are available and were superimposed to Amblyomin-X’s KD homology model (Figure 3).

Although TAP presents structural elements related to members of the Kunitz serine protease inhibitor family, its structure determination highlighted significant differences in the predicted binding site to FXa (loop L1 for Amblyomin-X, Figure 2A), in comparison to BPTI, suggesting a unique mode of binding to the enzyme (Lim-Wilby et al., 1995). Indeed, using structural analysis of bovine FXa complexed with TAP (Wei et al., 1998), and inhibition kinetics studies of wild type and human FXa mutants (Rezaie, 2004), it was demonstrated that TAP interacts with FXa mediated mainly by its N- and C-terminus, utilizing the active site and the autolysis loop of the enzyme. In comparison with TAP, Amblyomin-X’s KD also presents major structural differences in the loops L1 and L2 and the dissimilarities between these molecules are extended to their surface charge distribution, including their N- and C-terminus (compare Figures 3B,C, for Ixolaris and Amblyomin-X, respectively). Therefore, though it lacks experimental confirmation, it is unlikely that Amblyomin-X’s KD interacts with FXa the same way TAP does.

Ixolaris is composed of two KDs arranged in tandem and is the first inhibitor described to specifically target FXa heparin-binding exosite (HBE) (Monteiro et al., 2005). The second KD of Ixolaris, K2, is atypical, presenting just two disulphide bridges and several anionic amino acids, which were assumed to contribute for Ixolaris–FXa HBE interactions (Monteiro et al., 2005). In agreement, Ixolaris’ structure was recently solved (De Paula, et al., 2019) and the study revealed a noncanonical mechanism of binding to FXa HBE, with a major contribution from K2, shown to be conformationally dynamic, but it was also shown that the interaction requires both K1 and K2 domains (De Paula, et al., 2019).

The KD of Amblyomin-X is more similar to Ixolaris K2 (Figure 3D; 3F and 3H for individual comparison), also presents several anionic amino acids (Figures 2C, 3B), but it is predicted to have all of the three expected disulfide bridges and, therefore, structural rigidity. Amblyomin-X’s KD presents just one residue relevant to Ixolaris K2 interaction with FX/FXa structurally conserved, Y94 (De Paula, et al., 2019), although located in a patch of 4 identical residues. The comparison of Amblyomin-X’s KD to Ixolaris K1 reveals, again, just a single residue, structurally conserved, relevant to Ixolaris K1 interaction with FX/FXa, H57 (De Paula, et al., 2019), and major differences in the charge distribution profile. As speculated for TAP, it is unlikely that Amblyomin-X KD interacts with FXa the same way Ixolaris.

It is important to consider Amblyomin-X as a whole molecule, presenting an additional C-terminal portion near the size of its KD. Amblyomin-X’s C-terminal lacks homology to proteins in public databases, and it is assumed that it is potentially unstructured and possibly very flexible. Regarding Amblyomin-X’s inhibitory features, the C-terminal can potentially favor and/or modulate interactions in the whole molecule, which can’t be assessed by analyzing just the structured portion of the molecule. Additional experimental approaches will be used in order to clarify the contribution of the C-terminus to Amblyomin-X activities.

Hence, it is reasonable to assume that Amblyomin-X is a peculiar molecule that carries a particular KD at the N-terminal and a unique C-terminal. At the moment, the elements regarding protease inhibition and antitumoral mechanisms of action are still under investigation.

## Amblyomin-X activity

### Anticoagulant activity of amblyomin-X

Hematophagous animals contain several molecules with inhibitory activity towards serine proteinases or coagulation system complexes, in addition to vasodilators and platelet aggregation inhibitors (Arcà and Ribeiro, 2018; Chudzinski-Tavassi et al., 2018). These molecules help blood-sucking animals overcome the host’s defense mechanism to keep feeding on blood. Differences in blood-feeding behavior, especially in ticks, are reflected in the composition of anti-hemostatic toxins present in tick saliva. Soft ticks (family Argasidae) are fast feeding (less than 1 h), whereas hard ticks (family Ixodidae) feed for days or weeks, meaning they need more toxins to control the host’s immune and hemostatic systems (Šimo et al., 2017).

*A. sculptum* (former *A. cajennense*) is classified as a hard tick and is one of the most studied because of its ability to transmit the bacterium *Rickettsia rickettsii*, which causes the Brazilian spotted fever (BSF), the most lethal rickettsiosis in Brazil (Szabó et al., 2013). In addition, the saliva of this species yields many components that affect animal hemostasis and the immune system to ensure feeding for long periods (Chudzinski-Tavassi et al., 2018). From a cDNA library of the salivary glands of *A. sculptum*, several transcripts that interfere with the hemostatic system were identified among the 1754 clones analyzed, especially five types of serine protease inhibitors, reinforcing their involvement in the blood-feeding process (Batista et al., 2008, 2010; Jmel et al., 2021). Amblyomin-X has been identified in the cDNA library of *A. sculptum* salivary glands (Batista et al., 2010).

The recombinant Amblyomin-X was expressed in *E. coli* BL21 (DE3) as a 13.5 kDa inhibitor composed of a single Kunitz-type homologous domain in the N-terminal and a C-terminal with no similarity to any protease inhibitor or other sequences (Batista et al., 2010; Mesquita Pasqualoto et al., 2014). Like other inhibitors from ticks, Amblyomin-X’s Kunitz-domain shares structural similarity with TFPI, a physiological inhibitor of activated coagulation FXa. TFPI inhibits the extrinsic tenase complex formed by TF/active factor VII (FVIIa) through its first Kunitz-domain and FXa through its second Kunitz-domain (Chudzinski-Tavassi et al., 2016; Mann et al., 2019).

Amblyomin-X inhibited the hydrolytic activity of FXa, as determined by a chromogenic assay using a synthetic substrate for FXa. In addition, this inhibitor prolonged global blood clotting times, including activated partial thromboplastin time (APTT), prothrombin time (PT), and procoagulant activity (PCA), in assays using human plasma (*in vitro*). In *ex vivo* experiments, PT and APTT in Amblyomin-X-treated mice were not altered at doses of 1 mg/Kg, although a higher concentration of the inhibitor (2 mg/Kg) altered APTT (Batista et al., 2010). Conversely, PT and APTT in Amblyomin-X-treated rabbits were prolonged but reversibly (Branco et al., 2016). Kinetic studies showed that Amblyomin-X is a non-competitive FXa inhibitor, with a Ki of 3.9, and it is also capable of inhibiting approximately 50% of the tenase and prothrombinase complexes in *in vitro* assays, both at concentrations of 3 µM. Furthermore, Amblyomin-X is a substrate for plasmin and trypsin but not FXa and thrombin (Branco et al., 2016).

### Antitumor activity of amblyomin-X

Nowadays, it has been recognized that there is a mutual association between cancer and blood coagulation disorder. TF, the primary initiator of coagulation, is highly expressed in many types of malignancies (van den Berg et al., 2012). TF/FVIIa complex acts into protease activated receptor 2 (PAR-2) (Schaffner and Ruf, 2009), which in turn activates a number of downstream signaling involved in the production of pro-angiogenic factors, immune-modulatory cytokine and growth factors that supports tumor cell migration and metastasis (Unruh and Horbinski, 2020). TF/FVIIa complex inhibitors, including TFPI, possess opposing functions by reducing tumor development and metastasis (Amirkhosravi et al., 2007; Bajou et al., 2008; Fayard et al., 2009; Lavergne et al., 2013; Wang et al., 2018). Studies have demonstrated that TFPIs exhibit antiangiogenic and antimetastatic effects *in vitro* and *in vivo* and described them as apoptosis inducers in tumor cells (Amirkhosravi et al., 2007). TFPI-2 is downregulated in aggressive cancers, such as breast cancer and glioma, and recombinant therapy or overexpression of this protein reduces tumor cell migration and invasion (Fayard et al., 2009; Lavergne et al., 2013; Wang et al., 2018). The proposed mechanisms for these effects against cancers are related to inhibiting metalloproteinase activity, maintaining extracellular matrix integrity, and impairing tumor invasion and angiogenesis *in vivo* and *in vitro* (Izumi et al., 2000; Yanamandra et al., 2005; Provençal et al., 2008; Ran et al., 2009).

Ixolaris and penthalaris from the tick *Ixodes scapularis* was the first class of TFPI-like inhibitors from hematophagous organisms. In contrast to TFPI, ixolaris and penthalaris do not bind to the FXa active site (Francischetti et al., 2002; Francischetti et al., 2004). These two inhibitors bind to FXa and FX that serve as scaffolds for inhibition of the TF–FVIIa complex (Francischetti et al., 2004; Monteiro et al., 2008). The second class of inhibitors, Ascaris-type inhibitors, is represented by the recombinant NAPc2 (Nematode Anticoagulant Peptide c2) from the hookworm Ancylostoma caninum (Lee and Vlasuk, 2003; Koh and Kini, 2009). Although rNAPc2 and Ixolaris share a similar anticoagulant mechanism in relation to scaffold requirements for inhibition of TF/FVIIa complex, NAPc2 uses a different exosite in FXa to inhibit TF-VIIa (Murakami et al., 2007). In fact, NAPc2 blocks the active site of FVIIa, while locking in FXa in a signaling active conformation on the ternary TF-FVIIa-FXa complex (De Paula et al., 2019). It is noteworthy that in contrast to FXa inhibitors, specific inhibitors of the extrinsic tenase complex (active factor VII/tissue factor, FVIIa–TF complex) are associated with anti-cancer and anti-metastatic activities (Hembrough et al., 2003; Carneiro-Lobo et al., 2009; Zhao et al., 2009). Ixolarix and NAPc2 have demonstrated to be a potent anticancer agent (Carneiro-Lobo et al., 2009; Zhao et al., 2009). Ixolaris was able to block tumor growth of the human cell model through inhibition of direct TF–FVIIa–PAR2 signaling as well as its anticoagulant activity (Carneiro-Lobo et al., 2012). Amblyomin-X demonstrated to be able to inhibit FX activation by FVIIa/TF tenase complex in a concentration-dependent fashion (Morais et al., 2014). The inhibitor could interact directly with the active site of the enzyme or could sterically prevent access of the substrate to the active site (Batista et al., 2008, 2010; Morais et al., 2014; Branco et al., 2016). Experimental assays and *in silico* analysis indicated that Amblyomin-X could be considered functionally related to the TFPI-like inhibitors (Batista et al., 2008, 2010; Morais et al., 2014; Branco et al., 2016).

Based on success studies of coagulation factor inhibitors from hematophagous organisms in tumor cell lines (Tuszynski et al., 1987; Hembrough et al., 2003) and due to Amblyomin-X’s similarity with TFPI, a viability assay was performed with several tumor cell lines to evaluate the antitumor activity of this molecule. Amblyomin-X elicited cell death in several tumor cell lines, especially in those derived from solid tumors (NPI - PI0406057-1, Brazil, 14/09/2009), but could not induce death in non-tumor cells (human dermal fibroblast: HDF, adult). Hence, the selective antitumor activity of Amblyomin-X has been explored in different cancer models.

In an *in vitro* assay using a human melanoma cell line (SK-Mel-28) and primary fibroblasts, Amblyomin-X induced time- and concentration-dependent death, cell cycle arrest, and apoptosis only in tumor cells. Amblyomin-X did not affect non-tumor cells, suggesting its selectivity for cancer cells. Furthermore, in an *in vivo* assay, Amblyomin-X treatment (1 mg/kg daily for 14 days) induced tumoral mass regression and a considerable reduction in the percentage of internal metastasis in murine melanomas generated with the B16F10 cell line. Metastatic nodules in the lungs, kidneys, and lymph nodes decreased by 60% in treated animals compared to the metastatic lesions distributed in the internal organs of the control group (Chudzinski-Tavassi et al., 2010). In addition, the same tumoral mass regression results upon treatment with Amblyomin-X were also observed in equine melanomas. Horse melanomas are spontaneous, encapsulated, and usually benign and are a suitable translational model. Amblyomin-X (1 mg/kg of the tumor mass) was intratumorally injected every three days for 28 days. The tumor volume evolution and clinical animal conditions were monitored over five months. In all cases, Amblyomin-X treatment reduced tumor volume by at least 75% or even led to the complete disappearance of the tumor mass at the end of the treatment period (Lichtenstein et al., 2020).

In addition to its cytotoxic activity, Amblyomin-X can regulate cell adhesion and migration of human tumor cells like TFPI and TFPI-2. However, unlike other Kunitz-type inhibitors, such as TFPI and TFPI-2, Amblyomin-X showed tumor cell specificity. Schmidt et al. demonstrated that Amblyomin-X reduced the motility of melanoma cells Sk-MEL-28 by simultaneously decreasing urokinase-type plasminogen activator receptor (uPAR) and small GTPase production and MMP-9 secretion, leading to disruption of the actin cytoskeleton and reduced cell migration (Schmidt et al., 2020). Another important function of Amblyomin-X in controlling metastasis is its effect on normalizing the hypercoagulable state. The hypercoagulable or prothrombotic state is a clinical disorder that increases the risk of excessive blood clot formation due to an abnormality in the coagulation system (Caine et al., 2002; Jiao et al., 2021). Cancer can confer a hypercoagulable state and is associated with metastasis progression and development (Caine et al., 2002; Jiao et al., 2021). Ventura et al. compared the action of heparin, a potent anticoagulant that inhibits intravascular arrest of cancer cells and affects metastasis (Ventura et al., 2013), with Amblyomin-X in a melanoma model to investigate the potential of the anticoagulant Amblyomin-X as a therapeutic agent for cancer treatment. The results demonstrated that Amblyomin-X, similar to the classic anticoagulant heparin, can affect tumor progression, and this effect was accompanied by changes in coagulation parameters (ATPP and PT) that brought them back to normal levels (Ventura et al., 2013). Amblyomin-X can decrease the procoagulant released by murine melanoma cells. However, unlike heparin, Amblyomin-X has a substantial pro-apoptotic effect on tumor cells.

The selective cytotoxicity of Amblyomin-X has also been described in other cancer models such as renal carcinoma (Akagi et al., 2012; Maria et al., 2013; de Souza et al., 2016), ependymoma (Pavon et al., 2019) and pancreatic adenocarcinoma cell lines (Chudzinski-Tavassi et al., 2010; Morais et al., 2016; Pacheco et al., 2016; Schmidt et al., 2020). In the renal carcinomas model, Amblyomin-X reduced the proliferation rate of renal carcinoma (RENCA) cells, promoted cell cycle arrest, and induced apoptosis in a dose-dependent manner. Amblyomin-X treatment causes an imbalance between pro- and anti-apoptotic Bcl-2 family proteins, dysfunction/mitochondrial damage, reactive oxygen species (ROS) production, caspase cascade activation, and proteasome inhibition (PI) and downregulates the expression of crucial proteins (cyclin D1, Ki67, and P-glycoprotein (Pgp)) involved in the aggressiveness and resistance of renal carcinoma (Akagi et al., 2012; Maria et al., 2013; de Souza et al., 2016). Corroborating this finding, an *in vivo* assay in a mouse renal orthotopic model demonstrated that Amblyomin-X treatment significantly inhibited metastasis formation, and histological analyses showed that Amblyomin-X cytotoxicity was restricted to the tumor area, reinforcing the selective anti-tumor effect of the *in vivo* treatment (de Souza et al., 2016); The same pattern has been observed in primary cell and intracranial xenograft models of pediatric anaplastic ependymomas (EPNs). Amblyomin-X treatment induced a series of intracellular events linked to cytotoxic effects, leading to tumor cell death in EPN primary cells and was more significant than cisplatin. In addition, the treatment did not decrease the viability of non-tumoral cells (stem cells - hAFSCs), and their original morphological characteristics were preserved. Likewise, the *in vivo* results were consistent with those of previous studies. The results demonstrated that after 21 days of daily treatment with Amblyomin-X (1 mg/kg), the EPN xenograft model displayed significant tumor mass regression compared to the control (Pavon et al., 2019). In pancreatic adenocarcinoma, the cytotoxic effects of Amblyomin-X have been observed in several tumor cell lines, such as Mia-PaCa-2, Panc1, AsPC1, and BxPC3 (Chudzinski-Tavassi et al., 2010; Morais et al., 2016; Pacheco et al., 2016; Schmidt et al., 2020). However, a xenograft still needs to be performed. These findings reinforce that Amblyomin-X’s cytotoxicity seems restricted to tumor cells, making this recombinant protein very attractive for anticancer therapy.

### Antiangiogenic activity of amblyomin-X

Due to its antitumor activity and structural similarity with TFPI, the effects of Amblyomin-X on angiogenesis were also explored. (Drewes et al., 2012). showed the inhibitory effect of Amblyomin-X on vascular endothelial growth factor A (VEGF-A)-induced angiogenesis. Topically applying Amblyomin-X (10 ng/10 ml or 100 ng/10 ml) on mouse dorsal skin every 48 h simultaneously with VEGF-A treatment significantly reduced VEGF-A-induced angiogenesis. *In vitro* experiments have shown that Amblyomin-X treatment inhibits VEGF-A-induced endothelial cell proliferation, delays the cell cycle, and reduces cell adhesion and tube formation (Drewes et al., 2012).

Concerning the tube formation inhibition effect, this work also showed that Amblyomin-X treatment reversed the VEGF-A-induced increase in platelet endothelial cell adhesion molecule-1 (PECAM-1) expression in endothelial cells. PECAM-1 expression levels are important markers in endothelial cell-cell junctions, indicating tube organization during new vessel formation (Privratsky and Newman, 2014). The effect of VEGF-A on PECAM-1 expression was dependent on gene synthesis, as visualized by enhanced mRNA levels. On the other hand, the inhibitory effect of Amblyomin-X was not dependent on reduced PECAM-1 gene synthesis, because mRNA levels were equivalent in VEGF-A-treated cells and VEGF-A plus Amblyomin-X-treated cells.

Another study designed a set of *in vivo* and *in vitro* assays to explore the molecular Amblyomin-X action mechanisms on endothelial cell functions during angiogenesis. Using a dorsal chamber model, this study showed that Amblyomin-X reduced angiogenesis without any biological or chemical stimulation, such as VEGF-A. Mice dorsal skin was topically treated with Amblyomin-X (10 ng/10 ml, 100 ng/10 ml, or 1000 ng/10 ml). The treatments were administered once a day, every two days, for a total of three applications. The local angiogenic effects of Amblyomin-X on dorsal subcutaneous tissue were measured by intravital microscopy and PECAM-1 labeling. The data obtained showed that topically applying Amblyomin-X significantly reduced the number of vessels in the subcutaneous tissue of mice compared to phosphate-buffered saline (PBS) treatment, corroborating the results of a previous study (Drewes et al., 2015).

The authors also investigated the effects of proteins on vascular permeability. Intradermally injecting Amblyomin-X (10 ng/site, 100 ng/site, or 1000 ng/site) did not affect the microvascular permeability measured by Evans blue extravasation into the tissue. Furthermore, treating t-End endothelial cells with Amblyomin-X (10 ng/ml, 100 ng/ml, or 1000 ng/ml) *in vitro* did not modulate vascular permeability inducer levels, such as nitric oxide (NO) and prostaglandin E2 (PGE_2_). These results indicated that Amblyomin did not affect vascular permeability or endothelial contractile mechanisms.

Moreover, the authors showed that Amblyomin-X (10 ng/ml and 1000 ng/ml) treatments reduced cell migration and adhesion in the Matrigel® matrix (10 and 1000 ng/ml) *in vitro*. Analysis of tube formation showed that Amblyomin-X treatment (100 ng/ml) reduced the organization of new vessels in Matrigel® in t-End cells. Treatment with Amblyomin-X (100 ng/ml) reduced the expression of adhesion molecules vascular cell adhesion molecule 1 (VCAM-1) and β3 integrin, with no alterations in PECAM-1 or β1 integrin expression levels.

Together, these findings highlight that Amblyomin-X has antiangiogenic properties that directly reduce neo-vessel formation and inhibit VEGF-A-induced angiogenesis. Amblyomin-X reduces endothelial cell migration and tube formation and modulates angiogenic adhesion molecules, such as VCAM, integrins, and PECAM (induced by VEGF). The molecular mechanisms underlying these effects and their roles in antitumor activity need to be explored and clarified.

## Biodistribution, pharmacokinetic, and pre-clinical evaluations of amblyomin-X

Amblyomin-X is a promising drug candidate for cancer treatment because of its antitumor potential. Before human clinical trials, new drug candidates must undergo preclinical animal studies to predict drug behavior in patients and ensure their safety profile (Zhang et al., 2011). Boufeur et al. evaluated the biodistribution and pharmacokinetic properties of Amblyomin-X upon administration in healthy female BALB/c mice (Boufleur et al., 2019). The treatment was administered intravenously because protein drugs can be hydrolyzed by stomach enzymes if administered orally (Bruno et al., 2013).

Amblyomin-X was observed in the plasma 15 min after injection and was detected for up to 60 min. However, it was not detected in plasma samples collected 24 h after repeated daily administration. The surface plasmon resonance assay showed that Amblyomin-X could not bind to albumin, corroborating its rapid clearance from plasma because albumin is the main protein responsible for drug transportation in the blood (Smith et al., 2010). In addition, Amblyomin-X was also identified in its complete primary structure in the thymus, lungs, heart, liver, kidneys, and spleen less than 1 h after injection, and it remained detectable to a lesser extent in the liver, spleen, and kidneys after 24 h. These data suggest that Amblyomin-X is rapidly distributed in tissues.

Since there were no signs of Amblyomin-X in the intestine, protein excretion was only investigated in the urine, in which peptides corresponding to Amblyomin-X fragments were detected, suggesting that Amblyomin-X is excreted in the urine. Together, these data showed that Amblyomin-X exposure to total body organs was low and that the protein was quickly eliminated from the body with a calculated AUC of 11,862 μg min ml^−1^. AUC values are expected to be higher in humans because small animals possess higher metabolic rates (Kuroda et al., 2008). Interestingly, the clearance profile is similar to TFPI, a protein related to Amblyomin-X (Palmier et al., 1992).

Amblyomin-X was tagged with a fluorophore and injected daily into healthy mice to evaluate *in vivo* protein accumulation. Imaging showed that despite an initial accumulation in the abdominal region, after 24 h, there was only a small quantity of the labeled protein in the region, concentrated in the bladder, indicating the complete elimination of the drug and no long-term accumulation. Along with Boufeur et al.‘s findings, Souza et al. found that animals with orthotopic kidney tumors presented a persistent colocalization of Amblyomin-X within the tumor stroma until three days after administration, demonstrating that the drug has an affinity towards tumors and that the presence of tumors can delay drug elimination from the body (de Souza et al., 2016).

This affinity could be derived from the high expression of TF. A number of cancer/stromal cells are known to highly express tissue factor and microvesicles contain active-TF, having an impact in cancer progression and increasing the risk of venous thromboembolism (Owens and Mackman, 2011; van den Berg et al., 2012; Unruh and Horbinski, 2020). Some basic studies propose the use of positron emission tomography (PET) and/or near-infrared photo-immunotherapy (NIR-PIT) in cancer imaging and therapy (Luo et al., 2017; Aung et al., 2018). The treatment of TF-expressing BxPC-3 cells, *in vitro* or *in vivo*, using anti-TF antibody conjugated with indocyanine green (ICG), followed by near-infrared photoimmunotherapy (NIR-PIT) of tumor lead to the death of cancer cells (Aung et al., 2018). This approach was also applied using Ixolaris, a specific TF inhibitor from *Ixodes scapularis* (Francischetti et al., 2002), in an orthotopic glioblastoma (GBM) model in mice treated with a technetium-99 (99mTc) radiolabeled-Ixolaris (Barboza et al., 2015). The authors proposed ^99m^Tc-ixolaris as a radiopharmaceutical agent for TF-expressing cancers. It is plausible that the Amblyomin-X accumulation in tumors *in vivo* models is due to the affinity for TF, considering the structural similarity of Amblyomin-X with TFPI, which inhibits the extrinsic tenase complex formed by TF/active factor VII (FVIIa) through its first Kunitz-domain (Chudzinski-Tavassi et al., 2016; Mann et al., 2019).

With its biodistribution and pharmacokinetics well established, Durvanei and collaborators carried out a preclinical study to evaluate Amblyomin-X toxicity in healthy mice treated with different acute and subacute doses (Maria et al., 2018), following the guidelines from the Brazilian Regulatory Agency (ANVISA, 2013). Doses were defined based on our previous study (de Souza et al., 2016). After intravenous treatment, they evaluated general animal behavior, bodyweight variation, water and food consumption, mortality, and biochemical, hematological, and histopathological parameters.

Since concentrations higher than 256 mg/kg led to acute toxicity with high mortality rates (>50%) after 24 h, the lethal dose (LD50) was not determined. While there were no deaths in the acute dose group treated with a dose range of 0.25 mg/kg to 256 mg/kg, doses higher than 64 mg/kg promoted alterations in motor and sensorial signals that lasted until day six after treatment. Animals in the subacute dose group treated with 1 mg/kg of Amblyomin-X also showed motor alterations that disappeared after treatment (Maria et al., 2018). Compared to bortezomib, a commercial proteasome inhibitor drug approved by the Food and Drug Administration (FDA) for treating multiple cancers (de Bettignies and Coux, 2010), Amblyomin-X showed lower toxicity (Richardson et al., 2005; Rosiñol et al., 2005), with most adverse effects being reversible (Maria et al., 2018).

None of the groups showed any irreversible body mass loss. Despite a slight decrease in platelets and leukocytes in the acute dose group after 24 h of treatment, which was reversed after day 14, there were no alterations in blood cell morphology, coagulation, or PT in either group. Alterations in platelet and leukocyte counts observed at the acute dose were also observed upon bortezomib treatment (Lonial et al., 2008; Satoh et al., 2011). While Amblyomin-X treatment did not alter urea, creatinine, aspartate aminotransferase (AST), and alanine transaminase (ALT) levels, altered hepatic enzyme levels were observed with bortezomib treatment (Rosiñol et al., 2005), demonstrating the superior safety profile of Amblyomin-X.

Finally, histological analysis of the internal organs (kidneys, liver and spleen) showed no alterations upon Amblyomin-X treatment, except with the 256 mg/kg dose after 24 h of treatment, which was reversed after 14 days. These results enabled the determination of the maximum tolerable dose and the dose with no observed adverse effects, 16 mg/kg and 0.57 mg/kg, respectively, showing that Amblyomin-X is a promising drug with low toxicity and reversible side effects (Maria et al., 2018).

## Molecular mechanisms of amblyomin-X in tumor cells

In 2010, Chudzinski-Tavassi et al. conducted one of the first studies to explore the molecular mechanism by which Amblyomin-X drives tumor cell death. In this study, microarray analysis showed that 24 genes were modulated in human melanoma (SK-MEL-28) and human pancreatic adenocarcinoma (Mia-PaCa-2) cells after treatment with Amblyomin-X. Among these genes, the most upregulated was dynein cytoplasmic 1 light intermediate chain 2 (DYNC1LIC2), followed by proteasome beta-type subunit 2 (PSMB2) (Chudzinski-Tavassi et al., 2010). Amblyomin-X inhibits the proteasome, preferentially impending proteasomal trypsin-like activity (Chudzinski-Tavassi et al., 2010; Maria et al., 2013; Pacheco et al., 2016, 2014). Amblyomin-X-mediated PI and the increased polyubiquitinated protein pool were observed only in tumor cells, reinforcing the hypothesis that it might present tumor-cell-specificity *in vitro* and likely *in vivo*.

Proteasomes are multimeric proteolytic complexes responsible for degrading ubiquitinated proteins (Tanaka, 2009; Qu et al., 2021). Correct proteasome-mediated proteolysis is essential for activating or inhibiting cell signaling pathways involved in several cellular processes, including the cell cycle and apoptosis (Manasanch and Orlowski, 2017). The modulation of proteasome activity with specific inhibitors has emerged as a powerful strategy for cancer treatment (Manasanch and Orlowski, 2017). For instance, two proteasome inhibitors, bortezomib and carfilzomib (Crawford et al., 2011; Ruschak et al., 2011), have been approved by the FDA of the United States of America (United States) for treating refractory multiple myeloma, while several others are being clinically trailed (de Bettignies and Coux, 2010).

**FIGURE 4:**
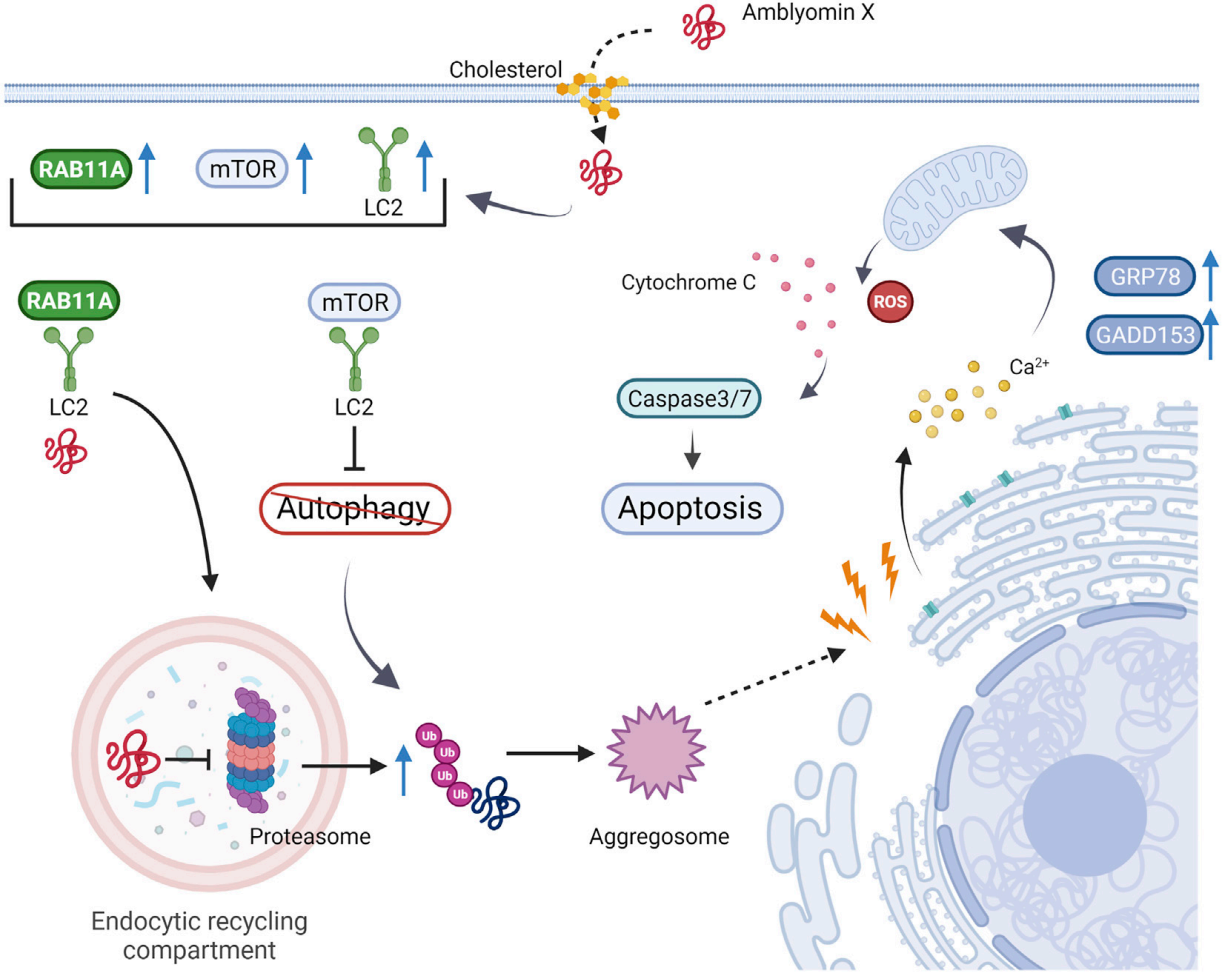
Mechanism of action of Amblyomin-X on tumor cells. The recombinant protein is internalized by endocytosis and transported by a dynein-assisted mechanism. Amblyomin-X inhibits proteasome activity, which results in aggresome formation via the non-exclusive ubiquitin pathway, ER stress, and mitochondrial dysfunction. Unlike other proteasome inhibitors, Amblyomin-X promotes dynein-assisted autophagy inhibition and mTOR localization. Therefore, the aggresomes are not cleared. These factors are crucial for apoptosis machinery to trigger a cell response. Figure created in BioRender.com.

Normally PI leads to the accumulation of protein aggregates in dynamic vesicles and aggresomes (Murphy, 2009), which activates the autophagy response to eliminate high molecular protein content. Dynein, a molecular motor that transports cellular components, is crucial in eliminating cytotoxic aggresomes after PI (Murphy, 2009). As mentioned above, Amblyomin-X positively modulates gene and protein expression of distinct dynein subunits (Chudzinski-Tavassi et al., 2010; Pacheco et al., 2014). In contrast to other known proteasome inhibitors, which require dynein only for aggresome and autophagic component transport (Kubiczkova et al., 2014), Amblyomin-X depends on a specialized uptake mechanism assisted by dynein for its inhibitory proteasome activity (Pacheco et al., 2016). Pacheco et al. demonstrated that cholesterol, phosphoinositide-3 kinase, and dynein are essential for Amblyomin-X internalization and transportation in tumor cells (Pacheco et al., 2016). Amblyomin-X action in tumor cells was abolished in cells pretreated with ciliobrevin, a small-molecule inhibitor of dynein ATPase activity and the Hedgehog pathway. In addition, the authors reported that Amblyomin-X not only interacted with dynein but also induced Rab11A overexpression and its colocalization with the light-intermediate chain 2 (LIC2) of dynein. Rab11A belongs to the Rab family of the small GTPase superfamily and is involved in recycling endosome trafficking *via* interaction with the LIC2 of dynein (Horgan and McCaffrey, 2009). Amblyomin-X was found in the perinuclear region where the endocytic recycling compartment was present, suggesting that recycling endosomes could be an Amblyomin-X intracellular destination in tumor cells (Pacheco et al., 2016). Moreover, when Pacheco et al. investigated dynein’s role in the proteasome-aggresome-autophagy pathway mediated by Amblyomin-X, they observed that the recombinant protein could induce aggresome formation *via* the non-exclusive ubiquitin pathway, and surprisingly, autophagy did not clear the aggresomes. In contrast to the available proteasome inhibitors, the presence of Amblyomin-X inhibited the autophagic response through mammalian target of rapamycin (mTOR) activation assisted by dynein transportation (Pacheco et al., 2016, 2014) (Figure 4).

Since PI results in the accumulation and aggregation of misfolded proteins, which can disturb homeostasis and lead to endoplasmic reticulum (ER) stress-related cell death (Ding et al., 2007; Deniaud et al., 2008; Hammadi et al., 2013; Cao and Kaufman, 2014), the effect of Amblyomin-X on ER stress was also evaluated. In RENCA cells, Amblyomin-X increased ER stress marker (GRP78 and GADD153) expression levels and altered [Ca^2+^] influx. Amblyomin-X can also induce mitochondrial dysfunction, marked by changes in its membrane potential and release of cytochrome c, provoking caspase-3 activation, ROS production, an imbalance between pro- and anti-apoptotic Bcl-2 family proteins, and tumor cell death *via* apoptosis (Maria et al., 2013). The same mechanisms of action for Amblyomin have been reported in human melanoma (SK-MEL-28) and pancreatic adenocarcinoma (Mia- PaCa-2) tumor cells. Amblyomin-X promoted pro-apoptotic effects associated with PI and ER stress, even in bortezomib-resistant (Mia-PaCa-2) tumor cells. Amblyomin-X inhibited proteasome function, ER stress, mobilization of (Ca^2+^), mitochondrial dysfunction, poly adenosine diphosphate-ribose polymerase (PARP) cleavage, and caspase-3 activation in tumor cells. Interestingly, few or no changes were observed in the normal human and mouse fibroblasts. Amblyomin-X did not induce PI, ER stress, or ROS production in fibroblasts, highlighting the selectivity of this molecule for tumor cells (Maria et al., 2013; Morais et al., 2016). Non-tumoral cell line (human dermal fibroblast: HDF, adult) cannot internalize Amblyomin-X, which requires a specialized uptake mechanism that seems to be restricted to tumor cells (Pacheco et al., 2016). The mechanisms governing tumor cell selectivity and internalization of Amblyomin-X are not completely understood and are still under investigation. One proposed hypothesis is that Amblyomin-X could present an affinity for exposed phosphatidylserine (PS) on the outer plasma membrane. PS is a negatively charged aminophospholipid that in healthy cells is normally found in the inner leaflet of plasma membrane (Yeung et al., 2008; Kay and Grinstein, 2011). In general, PS is exposed on the outer leaflet as a signal for phagocytosis in apoptotic cells (Fadok et al., 2000; Hoffmann et al., 2001) and also acts in processes such as myoblast fusion (van den Eijnde et al., 2001; Tsuchiya et al., 2018), immune regulation of non-apoptotic cells (Elliott et al., 2005; Fischer et al., 2006; Shlomovitz et al., 2019) and blood coagulation (Wang et al., 2022). In contrast, PS is often expressed at high levels on the outer leaflet of plasma membranes of viable cancer cells (Riedl et al., 2011; Chang et al., 2020). Preliminary findings by differential scanning calorimetry indicated that Amblyomin-X presents a preference for phosphatidylserine rather than phosphatidylcholine (date unpublished). Ongoing investigations will likely provide valuable information regarding the aspects involved in the tumor cell selectivity.

## Microenvironment and modulation of the immune response

Amblyomin-X is a protein that selectively leads to cell death by inducing ER stress, caspase activation, mitochondrial dysfunction, and PI in tumor cells (Chudzinski-Tavassi et al., 2010; Akagi et al., 2012; de Souza et al., 2016; Morais et al., 2016).

A recent study showed that this molecule could significantly reduce melanoma progression in horses (Lichtenstein et al., 2020). Mutations in driver genes cause malignant transformations. Horse melanomas are spontaneous and usually benign tumors, and a 4.6 kb duplication in intron 6 of *STX17* (syntaxin-17) causes the tumor phenotype, which constitutes a *cis*-acting regulatory mutation (Rosengren Pielberg et al., 2008). Mutated genes in human melanomas are *BRAF* and *NRAS*, as well as the newly discovered *PPP6C*, *RAC1, SNX31, TACC1, STK19*, and *ARID2* (Hodis et al., 2012). Although horse melanomas have a different natural history and many anatomical differences to human melanomas, they are a suitable translational model (van der Weyden et al., 2016).

*In vivo* treatment of encapsulated horse melanomas *via* intratumoral Amblyomin-X injections significantly reduced tumor size (Lichtenstein et al., 2020). Histological analysis revealed regression areas represented by the absence of atypical melanocytes in tumors treated with Amblyomin-X (Lichtenstein et al., 2020). Since melanomas are immunogenic tumors, the data analysis mapping and count resulted in 13,138 transcripts of valid gene symbols for horses and 13,943 for humans. For horse transcripts, 546 differentially expressed genes (DEGs) were identified for 6 h × 0 h and 259 DEGs for 12 h × 0 h, as modulated by Amblyomin-X and their respective cellular pathways (Lichtenstein et al., 2020). Enrichment analysis identified 196 and 67 pathways for 6 h × 0 h and 12 h × 0 h, respectively.

MetaCore analyses showed that the “immune system” was the class with the most enriched pathways, followed by the “innate immune system,” “inflammation,” “cancer,” and “adhesion/ECM/cytoskeleton” (Lichtenstein et al., 2020). Similar responses were found for 6 h × 0 h and 12 h × 0 h, including the “immune system”. Therefore, pathway classes were divided into three main groups: first response (ER stress and immune system), confounding factors, and secondary responses (Lichtenstein et al., 2020). For the immune system, some very important responses related to the innate immune system have been identified, such as the Toll-like receptor (TLR) signaling pathway, retinoic acid-inducible gene I (RIG-I)-like receptors (RLR), and oncostatin-M pathways. Besides this, apoptosis and cell death were inferred due to the enriched “ER stress” and “Apo-2L (TNFSF10)-induced apoptosis in melanoma” pathways (Lichtenstein et al., 2020).

According to Lichtenstein et al*.* (Lichtenstein et al., 2020), in silico crosstalk simulation between pathways identified five different possible crosstalks: “adhesion-ECM-cytoskeleton versus remodeling versus stress,” “immune cell death versus apoptosis versus ER-chaperone-Golgi,” “cancer versus complement versus inflammation,” “innate immune system versus apoptosis versus autophagy,” and “angiogenesis-vascular versus hypoxia versus stress”. Amblyomin-X also modulates important crosstalk between the innate immune system and apoptosis versus autophagy (Lichtenstein et al., 2020). It is possible to identify strong interactions between genes related to different biological functions, as genes participate simultaneously in various roles (Lichtenstein et al., 2020). In summary, the transcriptomic analysis showed that Amblyomin-X modulates several pathways, such as cytoskeleton remodeling, ER and mitochondrial dysfunction, tumor cell death, and the immune microenvironment (Lichtenstein et al., 2020).

Cellular interaction partners of Amblyomin-X were identified by interactome analysis, which showed that it potentially interacts with key transcriptomic elements (Lichtenstein et al., 2020). The equine melanoma cellular interactomics profile for Amblyomin-X was obtained by co-precipitation and showed multiple potentially interacting proteins in the eluent fraction that bound to immobilized Amblyomin-X. The partner proteins found are related to biological effects previously reported in Amblyomin-X studies, such as apoptosis, mitochondrial stress, cell cycle regulation, and proliferation (Chudzinski-Tavassi et al., 2010; Akagi et al., 2012; de Souza et al., 2016; Morais et al., 2016). This experimental approach using interactomics also identified immunogenic proteins, such as Toll-like receptor 2 (TLR2) and T-cell surface antigen CD2 (CD2), as possible Amblyomin-X interaction partners (Lichtenstein et al., 2020). Immunogenic cell death (ICD) constitutes a crucial pathway for activating the immune system against tumor progression, which determines the long-term success of anticancer therapies (Kroemer et al., 2013). Overall, these results suggest that Amblyomin-X can simultaneously orchestrate different pathways in a melanoma tumor model (Lichtenstein et al., 2020) by modulating the tumor immune microenvironment in different ways, leading to apoptosis and possibly ICD activation (Lichtenstein et al., 2020).

## Conclusion

Amblyomin-X is a recombinant protein derived from a cDNA library prepared from the salivary gland of the tick *Amblyomma sculptum*, which displays anticoagulant, antiangiogenic, and antitumor properties. Amblyomin-X presents a Kunitz-type domain that shares ∼40% similarity with the Kunitz domains of endogenous TFPI and Ixolaris, a well-known FXa inhibitor from *Ixodes scapularis*. Amblyomin-X’s cytotoxic activity in tumor cells and its ability to reduce tumor growth and metastasis in *in vivo* models has led to the vast exploration of its functions, action mechanisms, structures, pharmacokinetics, and biodistribution. Amblyomin-X drives tumor cell death *via* PI and appears to play a role in the tumor microenvironment by activating the immune system against tumor progression. A remarkable feature of this recombinant protein is that it seems to be restricted to tumor cells and does not affect non-tumorigenic cells, tissues, and organs, making it an attractive molecule for anticancer therapy, where the challenge is not only to suppress tumor growth but also to decrease treatment side effects. Therefore, considering the current efforts to develop effective anticancer therapies, Amblyomin-X could be a promising new antitumor drug candidate.
